# Evaluation and Monitoring of Endometrial Cancer Based on Magnetic Resonance Imaging Features of Deep Learning

**DOI:** 10.1155/2022/5198592

**Published:** 2022-03-18

**Authors:** Jingxiong Tao, Yi Wang, Yi Liang, Aohua Zhang

**Affiliations:** ^1^Department of Radiology, General Hospital of the Yangtze River Shipping, Wuhan Brain Hospital, Wuhan 430010, China; ^2^Department of Medical Imaging, 74th Army Hospital of PLA, Guangzhou 510318, China; ^3^Department of Ultrasound, The Third Affiliated Hospital of Sun Yat-Sen University, Guangzhou 510630, China

## Abstract

This study was aimed to compare and analyze the magnetic resonance imaging (MRI) manifestations and surgical pathological results of endometrial cancer (EC) and to explore the clinical research of MRI in the diagnosis and staging of EC. *Methods*. 80 patients with EC admitted to the hospital were selected as the research objects. The ResNet network was used to optimize the network. When the depth was added, the accuracy of the model was improved, the network parameters were iteratively updated, and the damage function of the minimized network was obtained. The recognition efficiency of MRI images was analyzed using three network modes: shallow CNN network, Res-Net network, and optimized network. The images of EC patients were analyzed, and a quantitative and timed MRI was achieved using simulated datasets in deep learning neural networks, which provided the basis for the formulation of single-scan MRI parameters. All patients underwent preoperative MRI examination using coronal and sagittal T1WI and T2WI imaging. The results showed that the accuracy and specificity of T2 weighted imaging and enhanced scanning in MRI were 88.75% and 95%, respectively. Sensitivity was 87.5%, negative predictive value was 93.75%, and positive predictive value was 86.25%. By MRI examination, 80 cases of EC in patients with stage I diagnosis were 72 cases, accounting for 90%, with endometrial thickening and uneven enhancement. In conclusion, the MRI manifestations of EC are diversified, and MRI has a high value for the staging of EC. MRI examination is conducive to improving diagnostic accuracy.

## 1. Introduction

Endometrial cancer (EC) is one of the most common malignant tumors in the female reproductive system. The incidence of EC is second only to cervical cancer, accounting for 15% to 20% of malignant tumors in the female reproductive system [1]. The essence of EC is a group of malignant tumors derived from the endometrial epithelial tissue. Most of the pathological types are endometrioid carcinoma, and other pathological types are mucinous adenocarcinoma, serous adenocarcinoma, and clear cell carcinoma [2, 3]. EC is more common in women aged between 50 years and 60 years, with an average age of 61 years, especially in people with obesity, menopause, hypertension, polycystic ovary syndrome, family history of cancer, and pelvic radiotherapy [4]. Symptoms mainly include vaginal bleeding, fluid discharge, abnormal vaginal secretion, uterine fluid, and pus [5, 6]. In women without menopause, symptoms include menorrhagia, prolonged menstruation or midmenstrual period bleeding, increased vaginal secretions (watery or bloody), and lower abdominal pain [7, 8]. EC is a common malignant tumor with a strong invasion in the female reproductive system. The treatment plan in the clinic needs to be determined according to the lesion and scope. The accurate staging of the lesion area is particularly important for the diagnosis of EC [9].

Conventional CT images are difficult to distinguish the lesion range, and it is difficult to distinguish stage II, stage III, and stage IV, and the judgment is limited to the intrauterine invasion [10]. Some “B” ultrasound for EC staging is not very accurate [11]. Magnetic resonance imaging (MRI) is widely used in clinical practice because of its good soft tissue resolution and high resolution, multidirectional, and high accuracy imaging characteristics. It is one of the highest imaging detection methods in the preoperative staging diagnosis of EC. The preferred method for the qualitative diagnosis of EC is diagnostic curettage. MRI diagnosis and differential diagnosis can detect other merger lesions [12]. MRI images are limited to qualitative measurement, and many research results cannot have a quantitative measurement result. The improvement of quantitative information has many significant advantages, such as parameter sensitivity, operation dependence, and spatial difference of magnetic field. These can be removed according to image requirements unrelated to tissue properties [13, 14]. Deep learning is widely used in computer computing. Its fast nonlinear mapping ability and ultrafast propagation ability have many applications in signal processing and many fields. Its application in image recognition, information processing, and model identification is also good. The operation of training a deep model has become simpler and easier [15]. However, the parameter data in MRI imaging are obtained for a long time, and the unconscious and conscious movement of patients will cause obvious artifacts.

Deep learning has made a series of breakthroughs and was widely used in speech processing, computer vision, natural language processing, and information retrieval [16]. The overlapping-echo detachment (OLED) method in deep learning includes the OLED quantitative T2 imaging with two overlapping echoes and the OLED quantitative T2 imaging with four overlapping echoes. The OLED technology is added to the single-scan quantitative T2 imaging, which can be used for the long parameter values in the region of interest (ROI), and the reconstruction effect is relatively poor. The OLED images with multiple echoes have stronger robustness in the long parameter values [17, 18]. Single-scan imaging needs further development, expands the perception field in the network of image imaging, and provides abundant quantitative information for image diagnosis. In this study, the U-Net network was used to analyze the images of patients with EC. The simulated data set was used in the deep learning neural network to achieve quantitative and timing MRI, which provided the basis for the development of single-scan MRI imaging parameters. The deep learning algorithm is introduced into the imaging to segment the MRI images better combined with the kernel space neighborhood information of the endometrial image. The characteristics of patients with EC are analyzed from different directions to provide a reference for the clinical diagnosis of EC, and the patients are treated effectively.

## 2. Methods and Materials

### 2.1. Case Selection

#### 2.1.1. Cases

Eighty patients with EC from the hospital were selected as the research objects. The patients were aged between 42 years and 70 years, with an average age of 51.67 ± 3.13 years old. The main types of diseases included 2 cases of carcinosarcoma, 32 cases of endometrioid adenocarcinoma, 6 cases of serous adenocarcinoma, 9 cases of low differentiation, and 10 cases of high differentiation. This study had been approved by the medical ethics of the hospital, and the patients or relevant people signed the informed consent form.

  Inclusion criteria [19]: (1) In line with the 2009 staging standard of EC of the International Federation of Gynecology and Obstetrics. (2) Patients with complete clinical data. (3) Independent cooperation with medical workers. (4) Patients who did not interrupt the treatment in the hospital. (5) Patients who met the treatment indications.
  Exclusion criteria: (1) Patients with severe diseases, such as organic lesions and hematopoietic system. (2) Patients with mental diseases. (3) Patients with poor treatment compliance. (4) Patients with communication barriers. (5) A variety of reasons lead to the interruption of treatment. (6) Tumors combined with other sites or metastatic tumors.

#### 2.1.2. MRI Examination Method

1.5 T MRI machine was used for pelvic MRI examination. The body coil was used as the RF emission coil, and the abdominal phased array wrapped coil was used as the receiving coil. Conventional coronal sagittal T2WI (fs FRFSE-XL, TR/TE = (3500–3800) ms/130 ms, NEX = 2–4, Fov = 26), cross-sectional T1WI (fs FSE-XL, TR/TE = 400 ms/8 ms, NEX = 2, Fov = 32), cross-sectional SE-EPI diffusion weighted imaging (DWI, *b* = 700, TR/TE = 4000 ms/73 ms) sequence, cross-sectional T2WI (fs FRFSE-XL, TR/TE = 4000 ms/130 ms, NEX = 4, Fov = 32), and LAVA cross-sectional masks were collected before MRI examination. Gd-DTPA was injected intravenously for multiphase dynamic contrast-enhanced scanning of the cross-section, followed by LAVA enhancement on the sagittal plane and coronal plane. Dynamic contrast-enhanced scan showed that the cervical mucosa showed epithelial discontinuous enhancement, which indicated cervical involvement. According to the diagnosis results, the patients were divided into a control group (without cervical infiltration) and an observation group (with cervical infiltration).

#### 2.1.3. Image Analysis and Diagnosis

Physicians analyzed the images of the patients according to the diagnostic criteria of the International Federation of Gynecology and Obstetrics for EC in 2009. The patients underwent MRI examination and were diagnosed by two experienced middle and senior professional doctors using a double-blind method. The endometrial thickness, cervical canal width, fiber matrix ring integrity, signal characteristics, and lymph node metastasis of patients were analyzed. Bilateral accessory and other organ invasions were observed. The MRI manifestations of EC with different invasion ranges are shown in Table 1, and the 2009 staging standard of EC of the International Federation of Gynecology and Obstetrics is shown in Table 2.

### 2.2. Convolution Neural Network Structure Model

**Figure 1:**
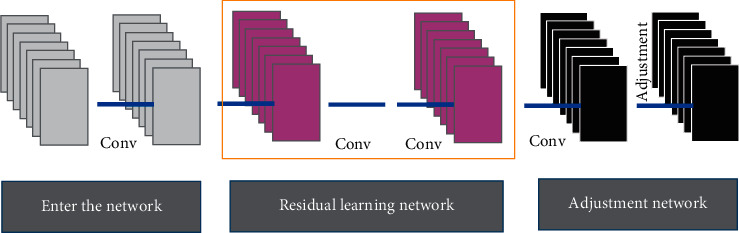
Convolution neural network structure model.

The characteristics of the training dataset play a very important role in the neural network. The training data set is too small to support the neural network training, however, the neural network in the excessive learning of the characteristics of the training samples will lead to the combination of training in the verification set, and the test set is not good enough, the generalization performance is not strong enough, and the phenomenon of excessive fitting occurs. Different training samples play a very important role in the reconstruction results. The adjustment network is a simple filter, and the output image is postprocessed. To reduce the noise in the image, the function of adjusting the network is to carry out slight denoising and guide the filtering operation to write to the last layer of the neural network, which can be reconstructed from end-to-end during the test. The filtering parameters do not need to be updated in the training stage. During the training process, the adjustment case is set as an identity mapping. The adjustment network is a guiding filter, and the guiding image is the image itself (Figure 1).

### 2.3. Network Structure Model

**Figure 2:**
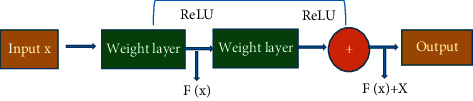
ResNet network.

A deep learning network is often used in medicine to learn the original image and is widely used in image segmentation, image classification, and target image localization. In the deep learning network, the pixel information at different scales can be combined to extract the optimal size information. The size of the convolution kernel can improve the operation speed of the neural network. The typical convolutional neural network model is used to reconstruct OLED. ResNet network generally avoids the disappearance of gradients in deeper networks. The deep abstract semantic information of the image can also be extracted. With the expansion of layers, different neural networks show different trends in the classification of accuracy in the model. For networks, such as AlexNet and GoogLeNet, classification accuracy is declining. The accuracy will increase with the increase of network depth to a certain extent, however, there will be a gradient explosion problem beyond this degree. Hence, the accuracy of the model training degree will decrease. There are residuals and residual modules in the NesNet network, and its accuracy is better than that of AlexNet, GoogLeNet, and other networks. The module connects the output with the input through skip connect (Figure 2). Each layer was input with a reference (X) to learn the formed residual function instead of learning some functions without reference (X). This residual function is easier to optimize and can greatly deepen the number of network layers. There were two layers in the residual block in Figure 1. The equation was expressed as follows:

(1) $$\begin{array}{c} F=W_{2}\delta \left(W_{1}X\right). \end{array}$$

*δ* represented the nonlinear function ReLU so that *Y* was obtained through a shortcut and the second ReLU.

(2) $$\begin{array}{c} Y=F\left(X\right)+X,\\ Y=F\left(X,\left\{W_{1}\right\}\right)+X. \end{array}$$

W was a convolution operation, which was used to adjust the channel dimension of *X*, normalize the initialization, and normalize the input of each layer so that the network depth that can converge was increased by 10 times.

In the convolution calculation, much image information of the input feature mapping of the corresponding residual module can be retained, which realizes that the NesNet network effectively improves the accuracy of the model when it is deeply added.

### 2.4. Optimized ResNet Network

**Figure 3:**
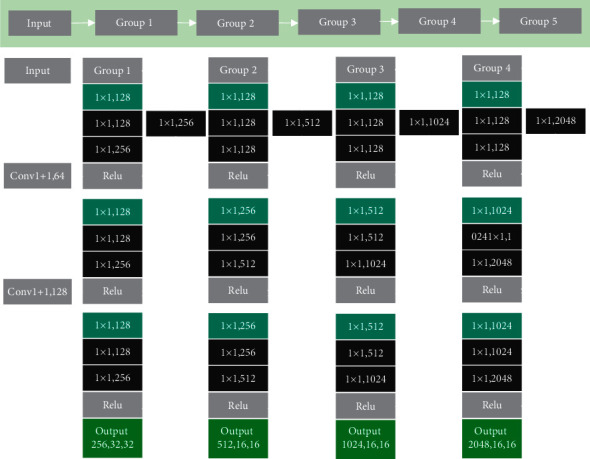
The structure diagram of optimized ResNet network.

The image recognition texture was optimized on the basis of ResNet network. In the first convolutional layer of the ResNet network, the function of the convolution kernel can increase the object receptive field, and the size of the image after preprocessing was 100 pixels × 100 pixels. On the basis of the original network, the step size in the first bottleneck residual result was all set to 1. Each layer of the network model used the ReLU activation function, adding the Adam optimizer (Figure 3).

### 2.5. Loss Function

The purpose of defining the loss function was to optimize the parameters of the neural network to represent the distance between the reconstructed image and the ground-truth label. The network parameters were iteratively updated according to the algorithm to minimize the loss function of the network and thus minimize the distance between the reconstructed image and the real sample. During the training of the network, the gradient descent method was used to gradually train and update the weighting coefficients and thresholds. The specific training steps were as follows:

Step 1: the neural network topology is initialized.
Step 2: the input sequence is input and read.
Step 3: calculation. The *H* and *Y* of hidden layer and output layer are calculated.
  (3) $$\begin{array}{c} \left\{\begin{array}{c} H_{j}=f\left(\sum_{i=1}^{n}\omega_{ij}-\theta_{j}\right)\\ Y_{k}=\sum_{j=1}^{l}H_{J}\omega_{jk}-\theta_{k} \end{array}.\right. \end{array}$$
*θj* is the hiding layer corresponding threshold, *θk* is the output layer corresponding threshold.
Step 4: operation. The mean square error (MSE) between the output value *Y* and the expected output value *Y* is calculated.
  (4) $$\begin{array}{c} E_{MS}\left(Y_{K}\right)=E{\left(Y_{k}-Y_{k}\right)}^{2}. \end{array}$$
Step 5: adjustment. The weighted coefficients of the hidden layer and output layer are as follows:
  (5) $$\begin{array}{c} \left\{\begin{array}{c} \omega_{ij}=\omega_{ij}+\eta H_{j}\left(1-H_{j}\right)x_{i}\sum_{k=1}^{m}\omega_{jk}E_{MS}\left(Y_{k}\right)\\ \omega_{jk}=\omega_{jk}+\eta H_{j}E_{MS}\left(Y_{k}\right) \end{array}\right.. \end{array}$$
*η* is learning efficiency.
Step 6: returning to Step 3 until error requirements are met.

Number of neuron nodes:

The optimal empirical equation is used to determine the range of values, and the minimum error corresponding to the number of hidden nodes is obtained after training. The empirical equation is as follows:

(6) $$\begin{array}{c} l=\frac{\text{n}\cdot \sqrt{\text{m}\cdot \text{n}}}{2}. \end{array}$$

The Sigmoid function is an activation function that can adjust the parameters to achieve precise control of the model. The expression is as follows:

(7) $$\begin{array}{c} S\left(x\right)=\frac{1}{1＋e^{-x}}. \end{array}$$

According to the principle of minimum weighting coefficient, the initial value and threshold of training samples are set.

The damage function defined in this study is a distance function. The goal of the network is to optimize the network parameters to minimize the distance between the reconstructed image and the true valued label. The equation is as follows:

(8) $$\begin{array}{c} L=\frac{1}{B}\sum_{\text{i}=1}^{B}{\left\|f\left(X\text{i},w,\xi \right)-Y^{i}\right\|}_{2}^{F}. \end{array}$$

In the equation, *N* represents the number of data in a batch, *X* is the input overlapping echo signal graph, *Y* is the true value label graph, *ξ* and *ω* represent the threshold and weight of the neural network to be learned, and *f*() represents the deep convolutional neural network.

To evaluate the model, the accuracy, sensitivity, and specificity were adopted to evaluate the prediction performance of the algorithm. The calculation equation was as follows:

(9) $$\begin{array}{c} Acc=\frac{TP+TN}{TP+TN+FP+FN}. \end{array}$$

(10) $$\begin{array}{c} \text{Sen}=\frac{TP}{TP+FN}. \end{array}$$

(11) $$\begin{array}{c} \text{Spe}=\frac{TN}{TN+FP}. \end{array}$$

In the above equations (9)–(11), *Acc* represented accuracy, *Sen* represented sensitivity, and *Spe* represented specificity.

### 2.6. Statistical Analysis

The survey data in this study were analyzed by SPSS19.0 statistical software. The measurement data conforming to the normal distribution were expressed as mean ± standard deviation $\left(\bar{\text{x}}\pm s\right)$, and the count data not conforming to the normal distribution were expressed as frequency (%). The *t*-test data was used for difference comparison, and chi-square test was used for quality comparison. When *P* < 0.05, the difference was statistically significant.

## 3. Results and Analysis

### 3.1. Experimental Data Distribution

The processor was Intel(R) Core (TM) i7-8750h CPU22.20GH, the hard disk size was 1T SSD, the programming language was Python 3.6.5, and the application program was CUPA/CUDNN 10.0.130, OpenCV-Python 4.1.0.25, Numpy1 .18.1, Matplotlib 2.2.2. The experimental data distribution was shown in Table 3. Compared with the shallow CNN network and the ResNet network, the results revealed that the optimized ResNet network showed the best performance.

### 3.2. MRI Images

**Figure 4:**
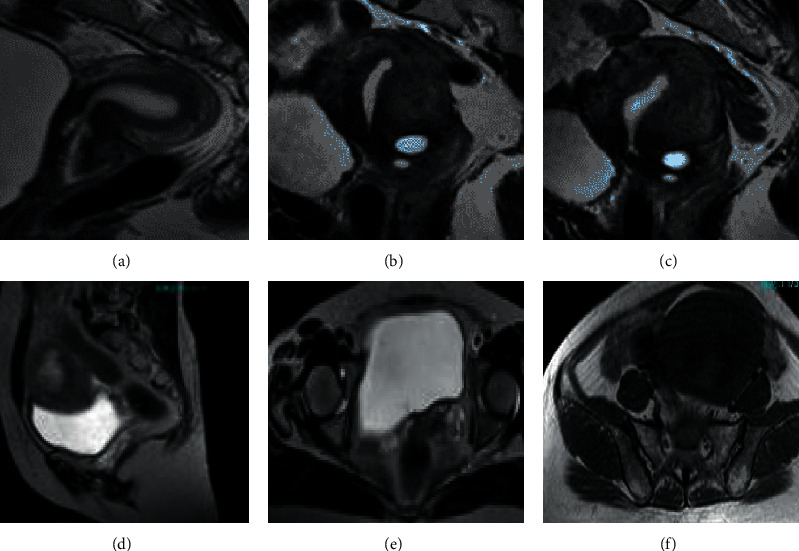
MRI images.

ABC is a normal endometrial MRI image. DEF is a 52-year-old female patient with early symptoms of large menstrual volume and intermittent abdominal pain for more than a year (Figure 4).

**Figure 5:**
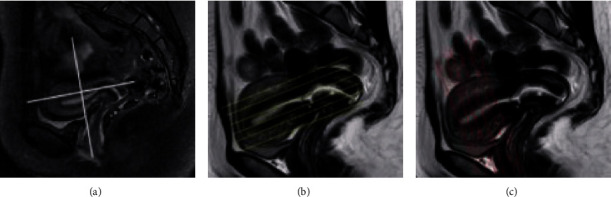
Scanning position images.

The general cases of EC include diffuseness and limitation, which are mainly divided into clear cell carcinoma, undifferentiated carcinoma, mixed carcinoma, squamous cell carcinoma, endometrial adenocarcinoma, and serous adenocarcinoma in histopathology. In Figure 5, figure 5(a) is the sagittal position, figure 5(b) is the coronal position, and figure 5(c) is the axial position. In Figure 5, the scanning direction is parallel to the uterine body long axis and perpendicular to the uterine body short axis.

**Figure 6:**
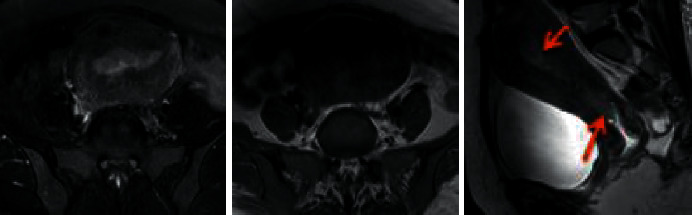
MRI image of cases.

Figure 6 shows a 50-year-old female patient with the main symptoms of incomplete menstruation for 13 days. The detection showed that the uterine cavity was hypoechoic and unevenly distributed. The EC was stage II, with medium-to-high differentiation. The depth of cancer infiltration was less than half of the muscular layer, involving the cervical canal. The MRI images in Figure 6 were from a patient with stage IIb endometrial cancer with dilated uterine cavity and thinning of the endometrial junction zone, as marked by the arrows.

### 3.3. MRI Diagnosis Results

**Figure 7:**
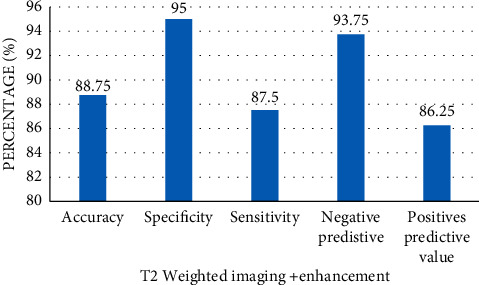
Ability of MRI to diagnose the depth of muscular infiltration.

For MRI T2 weighted imaging and enhanced scan, the accuracy was 88.75%, specificity was 95%, sensitivity was 87.5%, negative predictive value was 93.75%, and positive predictive value was 86.25% (Figure 7).

### 3.4. MRI Diagnosis and Pathological Control Results

As shown in Table 4, there were 72 cases of stage Ia in 80 patients with EC, 2 cases of stage III, 1 case of stage IV, 2 cases of stage Ib, and 3 cases of stage II in MRI diagnosis.

## 4. Discussion

EC is common in postmenopausal women. The main symptoms are postmenopausal bleeding and vaginal discharge, and 80% of the cases are EC [11]. MRI plays an important role in the diagnosis, staging, detection of curative effect, and detection of recurrence. The accuracy of MRI in the diagnosis of deep myometrium infiltration is 92% to 97%, and the accuracy of shallow myometrium infiltration is 69% to 74% [20, 21]. The measurement of uterine size is associated with many factors, such as uterine position, uterine fibroids, age, and hormone therapy history. The important high-signal area of the uterus shown on the sagittal T2WI in the MRI image mainly reflects the endometrial thickness in normal people. Patients with EC generally widen the uterine cavity, and the high-signal area widening in the central uterine body can be observed. It exceeds the upper bound of the normal range on the sagittal T2WI [22, 23]. The MRI detection method has the characteristics of parameter, nonradiation, and multiplanar imaging, and the image has good soft tissue resolution. It has a good effect in evaluating cervical and muscular invasion, extrauterine invasion, and lymph node metastasis of EC.

Diagnostic curettage is the preferred method for the qualitative diagnosis of EC. MRI can detect other lesions. Imaging must be used in the staging diagnosis of EC. Intrarectal ultrasound examination is suitable for staging that is still limited to the uterus. The accuracy of MRI is high, especially for stage I. In this study, MRI showed endometrial thickening in the diagnosis of EC, and T2WI showed slightly high signal intensity, which was lower than that of a healthy endometrium and higher than that of a muscular layer. Tsuyoshi et al. [24] used MRI to detect EC. The sensitivity, specificity, and accuracy of lesion-based detection of the regional lymph node metastasis were 100%, 96.9%, and 97.0%, respectively. MRI combined with positron emission tomography provides a high diagnostic value in evaluating primary tumors in patients with EC. MRI can provide a diagnostic strategy to replace conventional imaging. In this study, MRI was used to diagnose EC patients. The NesNet network was added in the imaging process to improve effectively the accuracy of the model. For MRI T2-weighted imaging and enhanced scan, the accuracy was 88.75%, specificity was 95%, the sensitivity was 87.5%, the negative predictive value was 93.75%, and the positive predictive value was 86.25%. Bi et al. (2019) [25] connected T2-weighted imaging, dynamic contrast-enhanced MRI, and diffusion-weighted imaging to realize the highest diagnostic accuracy and high specificity. MRI has good diagnostic performance for evaluating muscular infiltration in patients with EC. Dynamic contrast-enhanced MRI and diffusion-weighted imaging can improve the sensitivity and specificity of detecting myometrium infiltration. MRI has high specificity for detecting cervical infiltration and lymph node metastasis of EC. Fasmer et al. [26] recommended preoperative pelvic MRI for the local staging of EC, and the final tumor stage and grade were determined by surgery and pathology. The whole tumor radiological characteristics of MRI produce medium-high diagnostic performance, which is conducive to the assessment of preoperative risk for predicting invasive EC, and thus personalized treatment strategies are made for endometrial patients. The results of this study show that MRI has a high clear resolution and high accuracy for EC imaging, which has important clinical application value.

## 5. Conclusion

In this study, artificial intelligence is applied to the MRI imaging of medical EC patients, which effectively improves the efficiency of image feature extraction and classification of patients. Based on deep learning, intelligent algorithms were adopted to significantly improve the efficiency of diagnosis. Although the evaluation model of the deep learning algorithm has a certain reference value, there are still some shortcomings in practical application, for example, the experimental data cannot completely eliminate the interference of subjective factors. The next indicators can be standardized. In this study, the number of samples is small. The study is hoped to join more physiological and clinical data and further expand the sample in the future.

## Figures and Tables

**Table 1:** MRI manifestations of EC with different invasion ranges.

Invasion range	MRI appearance
Lymph nodes	Pelvic lymph node or para-aortic lymph node was greater than 10 mm in diameter
Vaginal bladder and rectum	Segmental disruption of normal signaling was replaced by that of tumor signaling
Shallow muscle layer	MRI showed partial or full-thickness disruption of junctional and subintimal enhancement bands, irregular intima-muscle interface, and no more than half of the muscle invasion
Deep muscular layer	MRI showed complete disruption of junction or subendometrial enhancement zone, tumor signal did not extend to more than 50% of the myometrium, and the myometrium infiltrated more than half of the uterus
Plasma membrane	MRI showed discontinuous outer edge of the myometrium and tumor beyond the contour of the uterus
Cervical mucosa	The internal cervical canal was widened by more than 3 mm, and the cervical fibrous stromal ring was intact
Cervical stroma	There were tumor signals in the cervical fibrous stromal ring
Limited to the endometrium	MRI showed normal or thickened endometrium (less than 5 mm after menopause and less than 10 mm before menopause), focal or diffuse abnormal signal, and intact and smooth subendometrial enhancement

**Table 2:** 2009 staging standard of EC

Staging	Features
Stage I	The tumor cells were found in the uterus only, and fat-suppressing T2W1 can accurately measure the depth of uterine tumor myometrial invasion
Stage Ia	The tumor cells were found in the endometrium or invaded no more than half of the myometrium only
Stage Ib	The tumor cells invaded more than half of the myometrium
Stage II	The tumor cells invaded the cervix without extracorporeal extension of the uterus
Stage IIa	The myometrium showed discontinues outer edge, and the tumor extended beyond the contour of the uterus
Stage IIb	This period was characterized by destruction of the cervical fibrous stromal ring
Stage III and Stage IV	The parametrial tissue and organ were affected with obvious changes in their signals
Stage III	The tumor cells invaded uterine serosa, adnexa, and vagina. Regional lymph node metastasis was found
Stage IV	The tumor cells invaded the bladder and rectum and metastased to distant organs

**Table 3:** Identification of MRI images using three models.

Type	Training set (piece)	Test set (piece)	Average identification time (piece)
Shallow CNN network	867	436	0.14 s
ResNet network	867	436	0.39 s
Optimized ResNet network	867	436	0.48

**Table 4:** MRI diagnosis and pathological control of EC.

Staging	Stage Ia	Stage Ib	Stage II	Stage III	Stage IV
Stage Ia shallow muscle layer infiltration	56	1	0	1	0
Stage II cervical infiltration	15	1	0	0	0
Stage III accessory vaginal involvement	0	0	3	0	0
Stage IV bladder and rectum involvement	0	0	0	1	1
Total	72	2	3	2	1

## Data Availability

The data used to support the findings of this study are available from the corresponding author upon request.
